# Identification of genes contributing to cisplatin resistance in osteosarcoma cells

**DOI:** 10.1002/2211-5463.13524

**Published:** 2022-11-29

**Authors:** Mingzhong Xie, Haoping Dai, Qingwen Gu, Changming Xiao, Haozhong Wang, Yang Lei, Chunxiao Wu, Xuening Li, Birong Lin, Sen Li

**Affiliations:** ^1^ The Affiliated Traditional Chinese Medicine Hospital of Southwest Medical University Luzhou China

**Keywords:** chemotherapy, cisplatin, genetic screen, osteosarcoma, *piggyBac* transposon, resistance

## Abstract

Osteosarcomas are prevalent in children and young adults and have a high recurrence rate. Cisplatin, doxorubicin, and methotrexate are common adjuvant chemotherapy drugs for treatment of osteosarcoma, but multidrug resistance is a growing problem. Therefore, understanding the molecular mechanisms of chemotherapy resistance in osteosarcoma cells is crucial for developing new therapeutic approaches and ultimately improving the prognosis of osteosarcoma patients. To identify genes associated with cisplatin resistance in osteosarcoma, we screened a large‐scale mutant library generated by transfecting human osteosarcoma cells with a *piggyBac* (PB) transposon‐based gene activation vector. Several candidate genes were identified by using Splinkerette‐PCR paired with Next Generation Sequencing. We created a disease‐free survival predictor model, which includes *ZNF720*, *REEP3*, *CNNM2*, and *CGREF1*, using TARGET (Therapeutically Applicable Research to Generate Effective Treatments) datasets. Additionally, the results of our enrichment analysis between the Four_genes_high group and Low_group suggested that these four genes may participate in cisplatin resistance in osteosarcoma through cross talk between various signaling pathways, especially the signaling pathway related to bone formation. These data may help guide future studies into chemotherapy for osteosarcoma.

## Graphical abstract

To identify genes associated with cisplatin resistance in osteosarcoma, a large‐scale mutant library was generated and several candidate genes were identified by using Splinkerette‐PCR paired with next‐generation sequencing. Additionally, a disease‐free survival predictor model, which includes ZNF720, REEP3, CNNM2, and CGREF1 using TARGET (Therapeutically Applicable Research to Generate Effective Treatments) datasets were created.

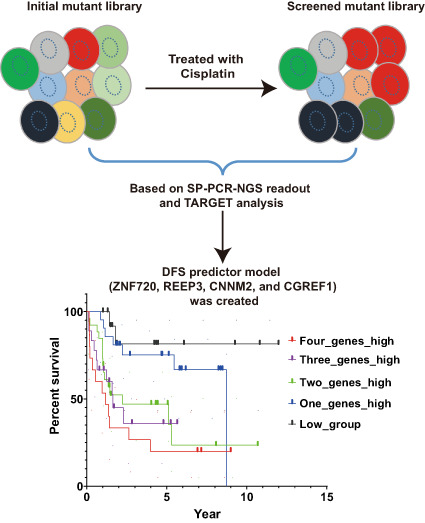

**Abbreviations**
BC: Bevacizumab‐containing chemotherapy
BMP4: bone morphogenetic protein‐4
BMPR1A: bone morphogenetic protein receptor type 1A
C0L: initial control library
C10L: Cis10_Screen_library
C5L: Cis5_Screen_library
CCND1: cyclin D1
CCNE1: cyclin E1
CGREF1: cell growth regulator with EF‐hand domain 1
CI: confidence interval
CNNM2: cyclin M2
CREOC: cisplatin‐resistant epithelial ovarian cancer
DEGs: differentially expressed genes
DFS: disease‐free survival
DKK1: Dickkopf Wnt signaling pathway inhibitor 1
EMT: epithelial‐mesenchymal transition
EOC: epithelial ovarian cancer
FOP: fibrodysplasia ossificans progressiva
FOS: FBJ osteosarcoma oncogene
GO: Gene Ontology
IBSP: integrin‐binding sialoprotein
IRGs: immune‐related genes
KEGG: Kyoto Encyclopedia of Genes and Genomes
K‐M: Kaplan–Meier
KRBOX5: KRAB box domain containing 5
MEF2C: myocyte enhancer factor 2C
PB: piggybac
PPI: protein–protein interaction
REEP3: receptor accessory protein 3
ROC: receiver operating characteristic
SD: splice donor
SOST: sclerostin
SP7: Sp7 transcription factor
SP‐PCR‐NGS: Splinkerette‐PCR paired with next generation sequencing
TARGET: therapeutically applicable research to generate effective treatments
TMEM119: transmembrane protein 119
TNIK: TRAF2 and NCK‐interacting protein kinase
ZNF720: Zinc finger protein 720, also known as KRBOX5

Primary malignant bone tumors in children and adolescents are predominantly osteosarcomas, prevalent in long diaphysis with high cellular heterogeneity. In recent decades, surgical resection combined with multi‐agent adjuvant chemotherapy has increased the 5‐year survival rate to 70–80% [1]. Cisplatin, doxorubicin, and methotrexate are the common adjuvant chemotherapy drugs [2, 3]. The 5‐year survival rate for patients with local recurrence and metastasis is < 20% despite increased doses or adjuncts to other agents and poor response to these agents [4]. Drug resistance and acquired resistance resulting from chemotherapy can limit its effectiveness and lead to cross‐resistance with diverse drugs [5]. Therefore, understanding the molecular mechanisms of chemotherapy resistance in osteosarcoma cells is crucial for developing new therapeutic approaches and ultimately improving the prognosis of osteosarcoma patients [6, 7].

The *piggyBac* (PB) transposon can move larger DNA fragments *in vitro* and has a significantly lower tendency toward local hopping. It has been used to discover mice cancer genes through insertional mutagenesis [8]. Two basic features of the PB‐based vectors used for insertional mutagenesis are loss‐of‐function and gain‐of‐function screens [9, 10]. Gain‐of‐function screens use a strong unidirectional exogenous promoter followed by a splice donor (SD) to initiate gene transcription regardless of whether the gene has been transcriptionally modified [9].

In this research, we aim to screen the cisplatin‐resistance factors generated through transfecting human osteosarcoma cells with the PB transposon‐based gene activating vector using a larger‐scale mutant library. After genetic screens, the candidate mutant genes can be identified using Splinkerette‐PCR paired with next generation sequencing (SP‐PCR‐NGS). Furthermore, a disease‐free survival (DFS) predictor model of osteosarcoma, including ZNF720, REEP3, CNNM2, and CGREF1, can be created using TARGET datasets, demonstrating that a combination of multiple genes may be responsible for cisplatin resistance.

## Materials and methods

### Cells and culture medium

The osteosarcoma cell lines (143B) were bought from the National Infrastructure of Cell Line Resource (NICLR, Beijing, China) and kept in our laboratory. The cells were cultured in Dulbecco's modified Eagle's medium (DMEM; Gibco, Shanghai, China) containing 10% FBS (HyClone, Logan, UT, USA). The cells were dissociated with 0.05% Trypsin (Gibco) at 37 °C for 3 min, harvested for passaging, and used in the following experiment. The cell numbers were detected using the Rigel cytometer (Countstar, Inno‐Alliance Biotech, Inc., Wilmington, DE, USA).

### Library construction and cisplatin screen

Approximately 1 × 10^7^ 143B were electroporated with 1 μg pPB‐CMV‐SD, and 20 μg pCMV‐PBase plasmids (Bio‐Rad Gene Pulser; Bio‐Rad Laboratories, Inc, Hercules, CA, USA) plated onto the 10 cm dish to create a large‐scale library. The medium containing puromycin (1.5 μg·mL^−1^) was replaced 48 h later and treated for an additional 7–8 days. Surviving cells were pooled as the PB transposon‐tagged library and stored at −80 °C for future experiments. The library was plated on a 10 cm dish for a Cisplatin screen and expanded into two passages. Then, the library was seeded into 3 × 10 cm culture dishes in 1 × 10^6^/dish and screened by adding 0, 5, and 10 μg·mL^−1^ cisplatin (S1166; Selleck Chemicals, Shanghai, China), respectively. The cells were treated for 10–14 days or until cisplatin‐resistant colonies were observed. Genomic DNA was extracted from the cells for NGS to obtain enriched genes.

### SP‐PCR paired with NGS and bioinformatics analysis

The methods of DNA library preparation and bioinformatics analysis was the same as that previously described [11]. Briefly, the Covaris S220 sonication system (Covaris, Woburn, MA, USA) was used to shear 10 μg of genomic DNA from each mixed library with fragment sizes ranging from 200 to 400 base pairs (bp). Following the manufacturer's instructions, DNA fragments were purified using AMPure XP beads. NEBNext Ultra II, DNA Library Preparation Kit for Illumina (E7645; NEB, Beijing, China), was used to repair and add 3′dA overhangs of these fragments, then ligate with Splinkerette linker. The junction fragments of PB3′ and PB5′ ITRs were amplified in two consecutive SP‐PCR rounds to generate PB3 and PB5 libraries, respectively. These libraries were sequenced on a single lane with pair‐end reads of 2*125 bases using Illumina HiSeq2500 at BGI (BGI Tech, Shenzhen, China). For bioinformatics analysis, a fastx toolkit (https://hannonlab.cshl.edu/fastx_toolkit/) was used to trim the reads of adapters and PB tags and followed by mapping to the human reference genome (hg38) using bowtie 2 software (http://bowtie‐bio.sourceforge.net/bowtie2/index.shtml).

### Bioinformatics' samples

Therapeutically Applicable Research to Generate Effective Treatments (TARGET) is an open database for childhood cancers. The raw count data of RNA‐sequencing (RNA‐Seq) and the relevant clinical information of 95 osteosarcoma samples in this study were obtained from the TARGET dataset (Table S2, https://ocg.cancer.gov/programs/target, phs000468). The requirements and application procedures were performed in compliance with relevant protocols and policies, are available at https://portal.gdc.cancer.gov/projects. For Kaplan–Meier (K‐M) curves, the survival analysis with *P*‐values, hazard ratio (HR) with a 95% confidence interval (CI) and a log‐rank test was chosen to compare the DFS between high‐ and low‐expression groups. The genes and their risk score were compared using time‐series receiver operating characteristic analysis (ROC). Multivariate cox regression analysis was used to construct a prognostic model, and the R package survival was used for the analysis. All R packages and analytical procedures were executed using r software (v4.0.3). Statistical significance was defined as adjusted *P*‐value < 0.01.

The differentially expressed genes (DEGs) were studied by using the limma package in R. “−log10 adjusted *P* > 2 and Log_2_ (Fold Change) > 1 or Log_2_ (Fold Change) < −1” were defined as the threshold for the differential expression of mRNAs. The data were analyzed using functional enrichment of Gene Ontology (GO) and Kyoto Encyclopedia of Genes and Genomes (KEGG) enrichment analysis to further confirm the underlying function of potential targets clusterprofiler package (version: 4.2.2) in r was employed to analyze GO and the KEGG. The ggplot2 package (version: 3.3.6) in r was used to draw the boxplot, and the pheatmap package (version: 1.0.12) in r was used to draw the heatmap. The protein–protein interaction (PPI) network and hub genes of DEGs were visualized using cytoscape version 3.7.0. The cytoHubba plugin was applied to identify the hub genes after the PPI network establishing.

## Results

### Large‐scale library of PB‐tagged osteosarcoma cells

**Fig. 1:**
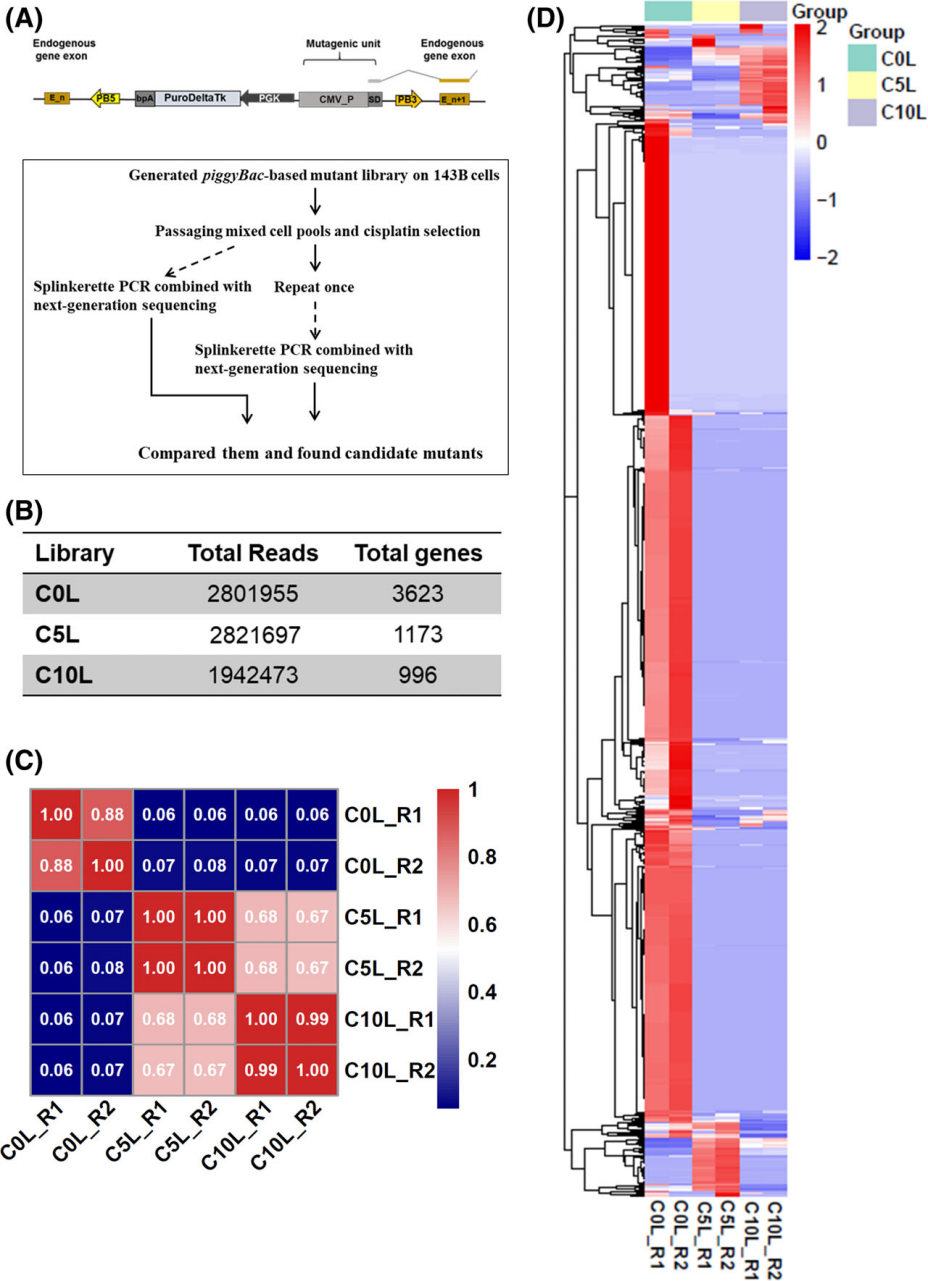
Generation and characterization of the screened libraries. (A) Top panel represents the structure of the PB vector for gene activation. The vector contains elements to elicit transcriptional activation (CMV and SD) and is shown integrated into an intron, with blue boxes representing exons of the gene that is activated. SD, splice donor; PB5 and PB3, PB5′ and PB3′ inverted terminal repeat; pA: SV40 ployA; CMV, Cytomegalovirus promoter. The bottom panel shows the genetic screening procedure. (B) Changes of the genes number in C0L, C5L and C10L. (C) A hierarchical clustering of gene distribution in C0L, C5L and C10L based on Pearson correlation coefficients. Blue to red indicates weak to strong correlations. (D) The heatmap showing the average transposon insertion frequencies in C0L, C5L, and C10L.

We establish a large‐scale PB transposon‐based gene‐activated library using the 143B osteosarcoma cells and PB transposon vector pPB‐CMV‐SD [12] to identify cisplatin‐resistance genes in osteosarcoma (Fig. 1A). The Mutant Library has been divided into three sub‐libraries. As the initial control library (C0L), 5 and 10 μg·mL^−1^ cisplatin were added, while Cis5_Screen_library (C5L) and Cis10_Screen_library (C10L) served as the screened libraries. Genomic DNA was extracted from three sub‐libraries, and the PB integration sites in mutant library cells were determined using SP‐PCR‐NGS (Fig. 1A). According to the deep sequencing data analysis, C0L covered 3623 mutant genes, C5L and C10L covered 1173 and 996 mutant genes, respectively (Fig. 1B). Pearson correlation coefficients (Fig. 1C) and heatmap (Fig. 1D) displayed significantly different patterns than C0L.

**Fig. 2:**
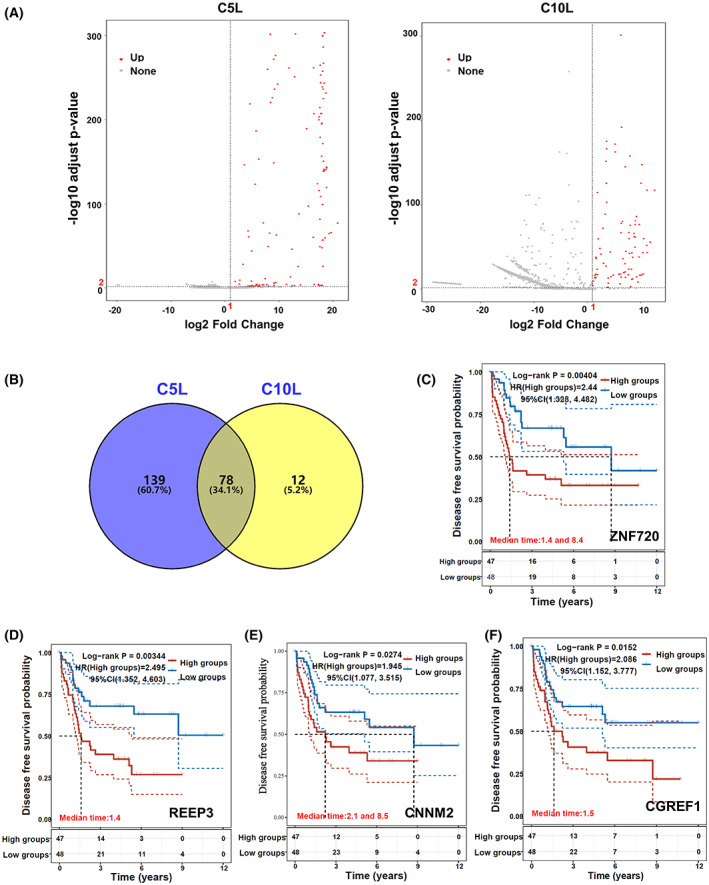
The gene overexpression was associated with DFS prognosis. (A) Red dots indicate enriched genes (log2FoldChange > 1, −log10 adjusted *P* value > 2), and gray dots indicate unchanged genes in MA plots for C5L and C10L. (B) Venn diagram depicting the overlapped genes among C5_Screen and C10_Screen libraries. A list of overlapped genes can be found in Table S1. (C–F) According to the median gene expression of ZNF720, REEP3, CNNM2 and CGREF1, the osteosarcoma patients in the TARGET dataset were grouped and each gene's DFS time (years) is revealed by Kaplan–Meier curves. The grouping is presented in Table S2.

Volcano plots of normalized mean reads versus fold change (Log_2_foldchange > 1, −log10 adjust *P*‐value > 2) revealed that 217 genes in the C5L and 90 genes in C10L were up‐regulated (Fig. 2A, Table S1). There were 78 overlapped genes between C5L and C10L (Fig. 2B, Table S1). Among these overlapped genes, some genes related to cisplatin therapy in other tumors have been reported. For example, malignant lung tumors with LncRNA SOX2‐OT, exhibit increased resistance to cisplatin‐based therapy and poor prognosis due to the modulation of ERK/AKT and SOX2/GLI‐1 expression [13]. In BET bromodomain inhibitor JQ1‐resistance HCT116 cells, which showed high resistance to various anti‐cancer drugs, including cisplatin, TNIK (TRAF2 and NCK‐interacting protein kinase) was a regulator of Wnt/β‐catenin signaling and transactivate cyclin D1 (CCND1) and cyclin E1 (CCNE1) [14]. The Semaphorin 4D (SEMA4D) has significantly higher positive expressions in cisplatin‐resistant epithelial ovarian cancer (CREOC) tissues with Bevacizumab‐containing chemotherapy (BC) response than in the BC nonresponse group, providing a novel therapeutic strategy and mechanism study for cisplatin‐resistant epithelial ovarian cancer (EOC) [15]. The osteosarcoma prognostic marker SOX2‐OT played an oncogenic role in osteosarcoma cell migration, invasion, and cancer stem cell biomarker expression [16]. TNIK has previously been identified as an essential factor for transactivating Wnt signal target genes, and its inhibition has been proven to eradicate colorectal cancer stem cells [17]. Several oncogenes, including SEMA4D and SEMA6D, have been involved in axon guidance in human osteosarcomas [18]. SEMA4D is one of the immune‐related genes (IRGs) used to generate clinical utility models [19]. These reports demonstrated that the enriched genes identified through our library screening might play an important role in osteosarcoma cisplatin resistance and our screening strategy efficacy.

**Fig. 3:**
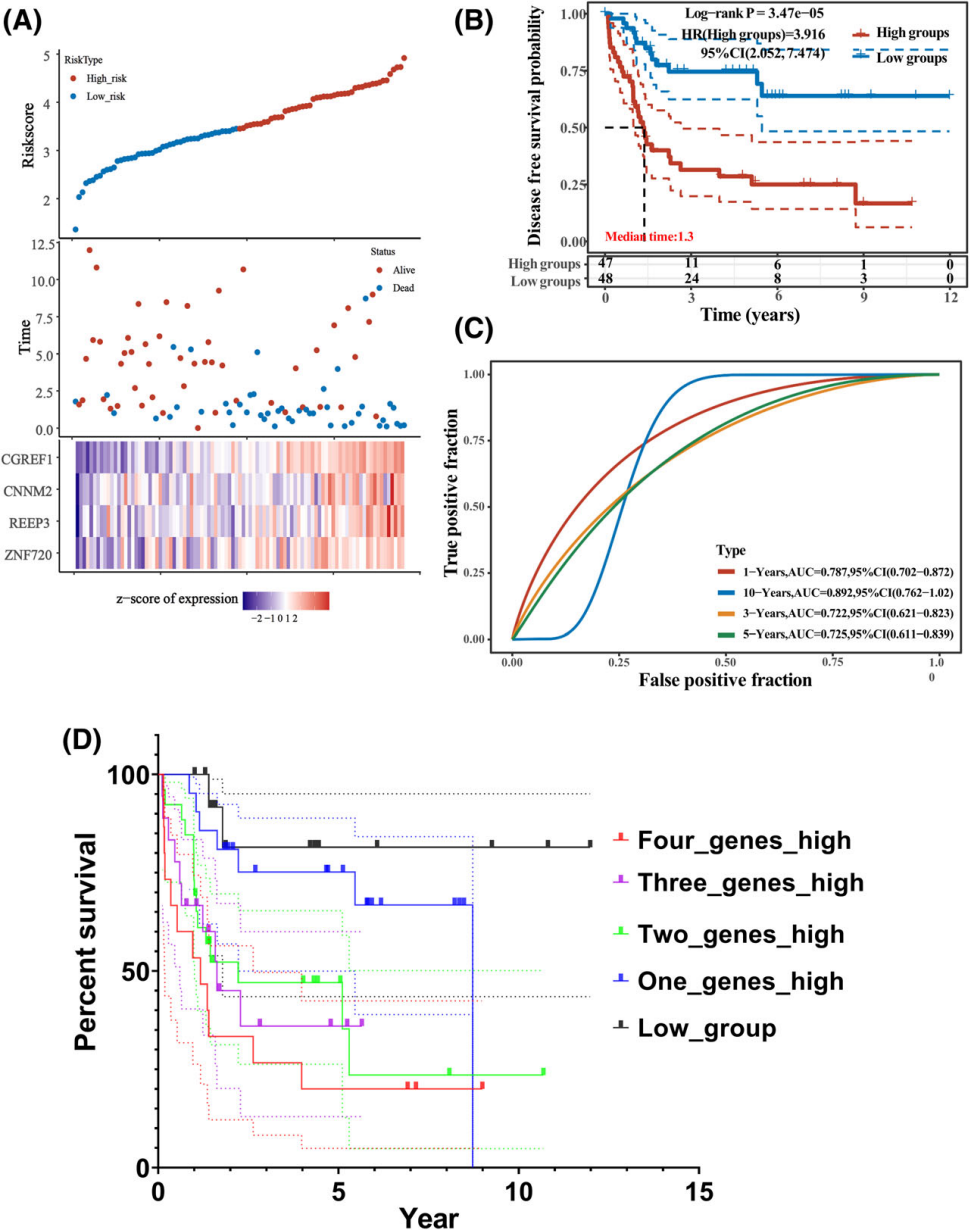
The gene signature as a relapse‐free survival DFS prediction model. (A) Prognostic analysis of the gene signature in the TARGET datasets, where the top represents the scatter diagram of the gene expression of median risk score from low to high and divided the patients into low‐risk (blue) and high‐risk (red) groups. The middle panel represents the scatter plot distribution of survival status and times responding to gene signature expression in different patient tumor samples. At the bottom, the heat map of the expression of the gene signature (CGREF1, CNNM2, REEP3 and ZNF720) was displayed. All the x‐axis of the top, middle and bottom of the three figures represent the 95 patient osteosarcoma tumor samples form TARGET dataset, and the order is consistent. (B) The Kaplan–Meier curve illustrates DFS time (years) in high‐expression and low‐expression groups of the gene signature according to the risk scores of these four genes. Among them, different groups were tested by log‐rank. HR (High EXP) represents the risk coefficient of the high‐expression groups compared with the low‐expression ones. HR > 1 indicates that the gene signature is a risk factor. 95% CI represents the HR confidence interval; Median time represents the corresponding median survival time, and the unit is the year. (C) The ROC curve and the area under the ROC curve (AUC) were used to assess the predictive accuracy of the gene signature. The higher values of AUC correspond to higher predictive power. (D) According to the median gene expression of ZNF720, REEP3, CNNM2 and CGREF1, the osteosarcoma patients in the TARGET dataset were divided into five groups (Four_genes_high, Three_genes_high, Two_genes_high, One_genes_high and Low_group). Kaplan–Meier curve was applied to assess the DFS time (years) between the five groups. Detailed information is provided about this figure can be found in Table S4.

The K‐M curve distribution of DFS prognosis‐related genes in the TARGET dataset showed that some overlapped enriched genes, such as Zinc finger protein 720 (ZNF720, also known as *KRBOX5*) (Fig. 2C), Receptor Accessory Protein 3 (REEP3) (Fig. 2D), cyclin M2 (CNNM2) (Fig. 2E) and Cell Growth Regulator with EF‐Hand Domain 1 (CGREF1) (Fig. 2F), were associated with osteosarcoma prognosis (Table S2). After calculating the risk scores (Table S3) of these four genes for each patient, they were divided into high‐ and low‐risk (Fig. 3A). K‐M survival analysis revealed a significant difference between the high‐ and low‐risk groups for DFS time (Fig. 3B). Akaike information criterion (364.0002) and risk score ((0.4193)*ZNF720 + (0.1222)*REEP3+ (0.1394)*CNNM2 + (0.291)*CGREF1) (Table S3) showed that these four genes (ZNF720, REEP3, CNNM2, and CGREF1) may be used to develop a relapse‐free survival DFS prediction model (Fig. 3C). A total of 94 osteosarcoma samples from the TARGET dataset were divided into five groups (Four_genes_high, Three_genes_high, Two_genes_high, One_genes_high, and Low_group) according to their expression levels in each sample to confirm further the correlation between these genes set with poor prognosis (Table S4). KM survival analysis showed that the Four_genes_high group had a poor DFS prognosis (Fig. 3D).

**Fig. 4:**
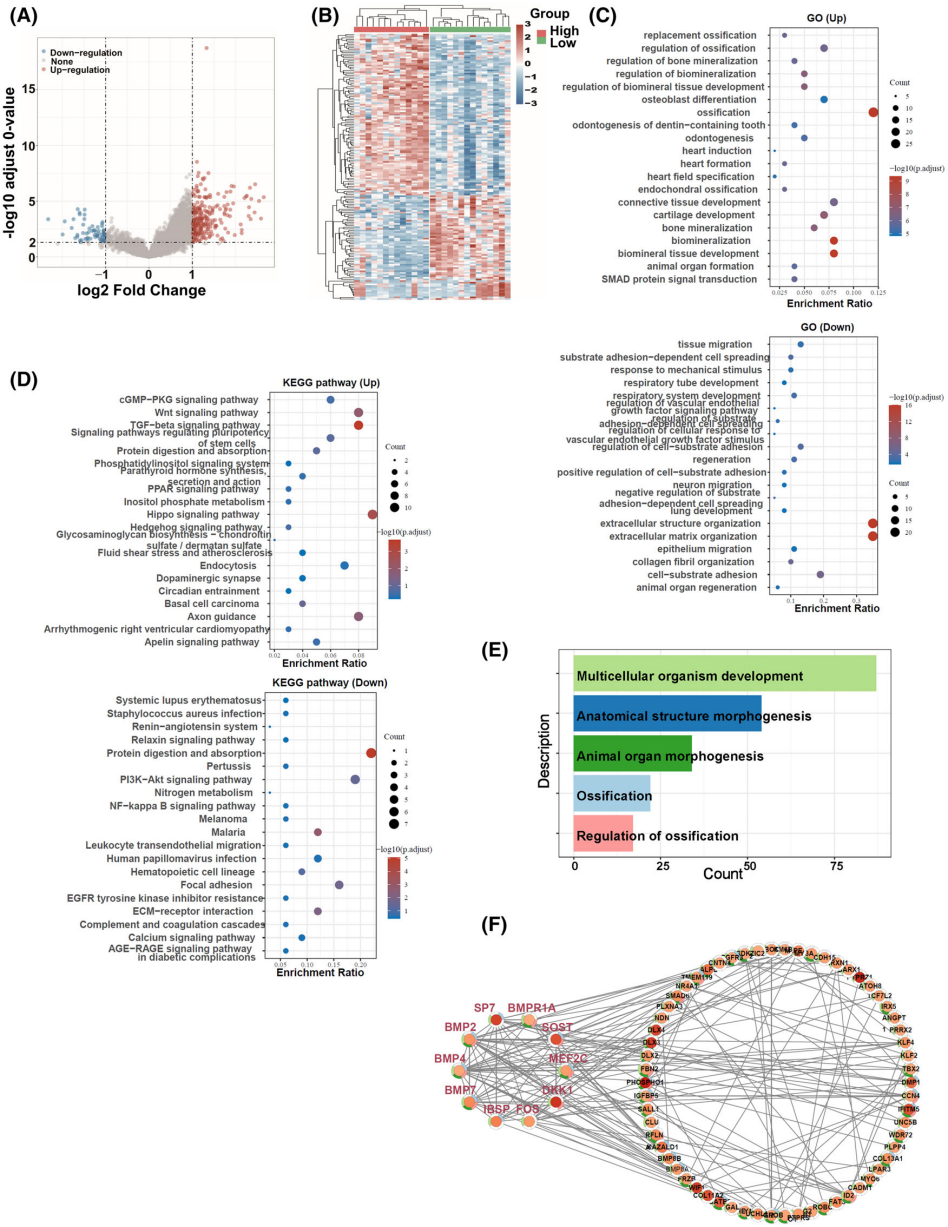
The enrichment analysis of the DFS prediction model. (A) Volcano plots presenting the 262 up‐regulation and 63 down‐regulation DEGs between the Four_genes_high group and Low_group. Red represents the up‐regulation genes (log2FoldChange > 1, −log10 adjusted *P* value > 2) and blue represents the down‐regulation genes (log2FoldChange < −1, −log10 adjusted *P* value > 2). (B) The heatmap showing the differential expression levels of the up‐regulated and down‐regulated genes in the patients of the Four_genes_high group and Low_group. (C) GO analysis of up‐regulated and down‐regulated enriched genes according to biological process. (D) The top 20 up‐and down‐regulated KEGG pathways are shown. (E) PPI network enrichment analysis construction up‐regulated pathways in Four_genes_high group compared with Low_group. (F) Ten hub genes and related genes in top 5 upregulated PPI pathways. The different colors in the rings correspond to different signaling pathways shown in E.

We carried out the enrichment analysis between the Four_genes_high group and Low_group to elucidate the mechanism underlying the poor DFS prognosis effect of the Four_genes_high group in osteosarcoma (Table S4). The Four_genes_high group exhibited a different expression pattern than Low_group (Table S5). DEG expression distributions, including 262 up‐regulated genes and 63 down‐regulated genes (Table S5), were shown (Fig. 4A,B). GO analysis indicated that ossification regulation, osteoblast differentiation, biomineral tissue development, and SMAD protein signal transduction‐related biological processes were up‐regulated (Fig. 4C) in the Four_genes_high group. In KEGG enrichment analysis, cGMP‐PKC, Wnt, TGF‐β, the pluripotency of stem cells regulation, Hipop, and Hedgehog (Hh) signaling pathways were more active (Fig. 4D). Cross‐talk between these pathways has been implicated in therapy resistance. According to PPI and cytoscape analysis, the top five GO biological pathways were Ossification, Anatomical structure morphogenesis, Multicellular organism development, Animal organ morphogenesis, and Regulation of ossification (Fig. 4E). The hub genes were Bone morphogenetic protein‐4 (BMP4), BMP2, BMP7, FBJ osteosarcoma oncogene (FOS), AP‐1 Transcription Factor Subunit, Sclerostin (SOST), SP7 (Sp7 Transcription Factor), Myocyte Enhancer Factor 2C (MEF2C), Bone Morphogenetic Protein Receptor Type 1A (BMPR1A), Integrin Binding Sialoprotein (IBSP), and Dickkopf Wnt Signaling Pathway Inhibitor 1 (DKK1) (Fig. 4F). These findings indicated that bone formation‐related genes and signaling pathways might participate in a high failure rate in osteosarcoma patients undergoing chemotherapy.

## Discussion

Cisplatin is a key drug in osteosarcoma therapy. It has been reported that several genes or pathways contribute to cisplatin resistance [19, 20, 21, 22]. In this study, we discovered that 78 genes might be responsible for cisplatin resistance using 143B osteosarcoma cells. A DFS predictor model, including ZNF720, REEP3, CNNM2, and CGREF1, demonstrated that a combination of genes might cause resistance to cisplatin. In previous reports, CNNM2, one member of the CNNM family, which contains four integral membrane proteins (CNNM1‐4), played a unique role in maintaining intracellular Mg^2+^ homeostasis and for its biological functions related to various cancers [23, 24]. REEP3 would possess the microtubule‐binding activity and remove the endoplasmic reticulum membrane from the metaphase chromosome [25]. The circFAT1 sponge miR‐30a‐5p regulates REEP3 overexpression, which could promote hepatocarcinogenesis [26]. ZNF720 is a novel transcript and also known as KRAB Box Domain Containing 5 (*KRBOX5*) and may regulate transcription [27, 28]. ZNF720 knockdown inhibited HIV‐1 replication in HeLa‐derived TZM‐bl cells [29]. CGREF1 is a novel secretory protein that regulated AP‐1 transcriptional activity and cell proliferation [30]. Although the above four genes have not been directly related to drug resistance to osteosarcoma, we speculate that they can predict drug resistance to osteosarcoma. Moreover, their mechanisms and roles in the chemo‐resistance of osteosarcoma need further study.

The PPI network and cytoscape enrichment analysis identified 10 hub genes, and five top GO biological pathways. BMP2 stimulated chondrogenic and osteogenic differentiation through BMPR1A [31]. BMP2 also enhanced osteosarcoma proliferation through Wnt/β‐catenin/epithelial‐mesenchymal transition (EMT) signaling pathway [32]. Further research showed that Transmembrane protein 119 (TMEM119) promoted osteosarcoma cell proliferation, migration, and invasion via increasing the TGF‐β pathway‐related factors (BMP2, BMP7, and TGF‐β) expression [33]. SP7, the human homolog of the mouse Osterix gene, was expressed in human fetal osteoblasts and craniofacial osteoblasts, chondrocytes, and osteosarcoma cell lines that required BMP2 induction [34]. ERK/JNK and c‐JUN/c‐FOS as upstream activators of FOXP1 drive osteosarcoma development by regulating the p53‐P21/RB signaling cascade [35]. FOS also efficiently initiated key stem cell proliferation, migration, division, and differentiation [36]. MEF2C and FOS can regulate the prognostic genes related to Osteosarcoma Metastasis [37].

In KEGG, cGMP‐PKC, TGF‐β, Hippo, Hh, and Wnt signaling pathways, as well as signaling pathways regulating pluripotency of stem cells, were more active in the osteosarcomas patients of the Four_high group. They were combined with five top GO biological pathways, including Multicellular organism development, anatomical structure morphogenesis, animal organ morphogenesis, and ossification regulation pathways. Over‐activation of the BMP signaling pathway can trigger heterotopic ossification in fibrodysplasia ossificans progressiva (FOP), a rare, progressive disease of massive HO formation [38]. PPI analysis Cross‐talk between these pathways may be important for therapy resistance. These findings suggest that these four genes may participate in osteosarcoma cisplatin‐resistance through various signaling pathways cross‐talks, especially the signal pathway related to bone formation.

Although activation of genes was associated with cisplatin‐resistance of osteosarcoma, this research has some disadvantages. First, we only identified a limited set of candidate genes due to the relatively low throughput of the library. Second, the behavior of drug‐resistant cells may differ when cultures are performed *in vitro* and *in vivo*. Therefore, *in vivo* experiments are necessary to verify our findings. Despite these potential limitations, our study could identify consistent gene expression information in osteosarcoma tumor cells. Additionally, we identified a new possible gene signature for osteosarcoma prognosis based on resistance genes. Therefore, our study should impact chemotherapy treatment for osteosarcoma patients.

## Conflict of interest

The authors declare no conflict of interest.

## Author contributions

SL conceived the project and designed the experiments; MX, HD and QG carried out the majority of the experiments and were the major contributor in writing the manuscript; CX, HW, YL and CW helped conduct and analysis the experiments; HD and BL performed bioinformatics analyses; SL, BL and XL provided critical review for the manuscript. All authors analyzed and discussed these results, reviewed and approved the submitted manuscript.

## Supporting information


**Table S1.** Overlapped enriched genes in C5_Screen and C10_Screen.

**Table S2.** The detailed data of DFS tines (years) in high‐ and low‐expression groups of ZNF720, REEP3, CNNM2 and CGREF1.

**Table S3.** The lambda parameter shows the coefficients of selected features. The abscissa represents the lambda value, and the ordinate represents the coefficients of the independent variable: The Risk score, survival time, and survival status of the selected dataset.

**Table S4.** The 96 osteosarcoma samples in the TARGET dataset were divided into five groups (Four_genes_high, Three_genes_high, Two_genes_high, One_genes_high, and Low_group) according to median gene expression levels of ZNF720, REEP3, CNNM2 and CGREF1 in each sample.

**Table S5.** DEG_results, DEG_genes and DEG_ Deg_clusterProfiler (PPI network, including KEGG and GO term) in Four_genes_high compared with Low_group.

## Data Availability

The data that support the findings of this study are available from the corresponding author (lisen_swmctcm@163.com) upon reasonable request. The data are not publicly available due to privacy restrictions.

## References

[1] Gianferante DM , Mirabello L , Savage SA . Germline and somatic genetics of osteosarcoma – connecting aetiology, biology and therapy. Nat Rev Endocrinol. 2017;13:480–91. 28338660 10.1038/nrendo.2017.16

[2] Xiao X , Wang W , Li Y , Yang D , Li X , Shen C , et al. HSP90AA1‐mediated autophagy promotes drug resistance in osteosarcoma. J Exp Clin Cancer Res. 2018;37:201. 30153855 10.1186/s13046-018-0880-6 PMC6114771

[3] Zhu KP , Zhang CL , Ma XL , Hu JP , Cai T , Zhang L . Analyzing the interactions of mRNAs and ncRNAs to predict competing endogenous RNA networks in osteosarcoma chemo‐resistance. Mol Ther. 2019;27:518–30. 30692017 10.1016/j.ymthe.2019.01.001 PMC6401193

[4] Mirabello L , Zhu B , Koster R , Karlins E , Dean M , Yeager M , et al. Frequency of pathogenic germline variants in cancer‐susceptibility genes in patients with osteosarcoma. JAMA Oncol. 2020;6:724–34. 32191290 10.1001/jamaoncol.2020.0197 PMC7082769

[5] Haven B , Heilig E , Donham C , Settles M , Vasilevsky N , Owen K , et al. Registered report: a chromatin‐mediated reversible drug‐tolerant state in cancer cell subpopulations. Elife. 2016;5:e09462. 26905833 10.7554/eLife.09462 PMC4775209

[6] Valencia AM , Kadoch C . Chromatin regulatory mechanisms and therapeutic opportunities in cancer. Nat Cell Biol. 2019;21:152–61. 30602726 10.1038/s41556-018-0258-1 PMC6755910

[7] Grunewald TGP , Cidre‐Aranaz F , Surdez D , Tomazou EM , de Alava E , Kovar H , et al. Ewing sarcoma. Nat Rev Dis Primers. 2018;4:5. 29977059 10.1038/s41572-018-0003-x

[8] Rad R , Rad L , Wang W , Cadinanos J , Vassiliou G , Rice S , et al. PiggyBac transposon mutagenesis: a tool for cancer gene discovery in mice. Science. 2010;330:1104–7. 20947725 10.1126/science.1193004 PMC3719098

[9] Kawakami K , Largaespada DA , Ivics Z . Transposons as tools for functional genomics in vertebrate models. Trends Genet. 2017;33:784–801. 28888423 10.1016/j.tig.2017.07.006 PMC5682939

[10] Stanford WL , Cohn JB , Cordes SP . Gene‐trap mutagenesis: past, present and beyond. Nat Rev Genet. 2001;2:756–68. 11584292 10.1038/35093548

[11] Pettitt SJ , Rehman FL , Bajrami I , Brough R , Wallberg F , Kozarewa I , et al. A genetic screen using the PiggyBac transposon in haploid cells identifies Parp1 as a mediator of olaparib toxicity. PLoS One. 2013;8:e61520. 23634208 10.1371/journal.pone.0061520 PMC3636235

[12] Collier LS , Carlson CM , Ravimohan S , Dupuy AJ , Largaespada DA . Cancer gene discovery in solid tumours using transposon‐based somatic mutagenesis in the mouse. Nature. 2005;436:272–6. 16015333 10.1038/nature03681

[13] Herrera‐Solorio AM , Peralta‐Arrieta I , Armas López L , Hernández‐Cigala N , Mendoza Milla C , Ortiz Quintero B , et al. LncRNA SOX2‐OT regulates AKT/ERK and SOX2/GLI‐1 expression, hinders therapy, and worsens clinical prognosis in malignant lung diseases. Mol Oncol. 2021;15:1110–29. 33433063 10.1002/1878-0261.12875 PMC8024737

[14] Takahashi C , Kondo S , Sadaoka K , Ishizuka S , Noguchi K , Kato Y , et al. Effect of TNIK upregulation on JQ1‐resistant human colorectal cancer HCT116 cells. Biochem Biophys Res Commun. 2020;530:230–4. 32828291 10.1016/j.bbrc.2020.06.136

[15] Zhang L , Chen Y , Li F , Bao L , Liu W . Atezolizumab and bevacizumab attenuate cisplatin resistant ovarian cancer cells progression synergistically via suppressing epithelial‐mesenchymal transition. Front Immunol. 2019;10:867. 31105696 10.3389/fimmu.2019.00867 PMC6498972

[16] Wang Z , Tan M , Chen G , Li Z , Lu X . LncRNA SOX2‐OT is a novel prognostic biomarker for osteosarcoma patients and regulates osteosarcoma cells proliferation and motility through modulating SOX2. IUBMB Life. 2017;69:867–76. 28960757 10.1002/iub.1681

[17] Hirozane T , Masuda M , Sugano T , Sekita T , Goto N , Aoyama T , et al. Direct conversion of osteosarcoma to adipocytes by targeting TNIK. JCI Insight. 2021;6:e137245. 33400690 10.1172/jci.insight.137245 PMC7934882

[18] Moriarity BS , Otto GM , Rahrmann EP , Rathe SK , Wolf NK , Weg MT , et al. A sleeping beauty forward genetic screen identifies new genes and pathways driving osteosarcoma development and metastasis. Nat Genet. 2015;47:615–24. 25961939 10.1038/ng.3293 PMC4767150

[19] Li J , Su L , Xiao X , Wu F , Du G , Guo X , et al. Development and validation of novel prognostic models for immune‐related genes in osteosarcoma. Front Mol Biosci. 2022;9:828886. 35463956 10.3389/fmolb.2022.828886 PMC9019688

[20] Lee AM , Ferdjallah A , Moore E , Kim DC , Nath A , Greengard E , et al. Long non‐coding RNA ANRIL as a potential biomarker of chemosensitivity and clinical outcomes in osteosarcoma. Int J Mol Sci. 2021;22:11168. 34681828 10.3390/ijms222011168 PMC8538287

[21] Wang Y , Deng X , Yu C , Zhao G , Zhou J , Zhang G , et al. Synergistic inhibitory effects of capsaicin combined with cisplatin on human osteosarcoma in culture and in xenografts. J Exp Clin Cancer Res. 2018;37:251. 30326933 10.1186/s13046-018-0922-0 PMC6192127

[22] Yang D , Xu T , Fan L , Liu K , Li G . microRNA‐216b enhances cisplatin‐induced apoptosis in osteosarcoma MG63 and SaOS‐2 cells by binding to JMJD2C and regulating the HIF1α/HES1 signaling axis. J Exp Clin Cancer Res. 2020;39:201. 32972441 10.1186/s13046-020-01670-3 PMC7517798

[23] Funato Y , Miki H . The emerging roles and therapeutic potential of cyclin M/CorC family of Mg(2+) transporters. J Pharmacol Sci. 2022;148:14–8. 34924118 10.1016/j.jphs.2021.09.004

[24] de Baaij JH , Hoenderop JG , Bindels RJ . Magnesium in man: implications for health and disease. Physiol Rev. 2015;95:1–46. 25540137 10.1152/physrev.00012.2014

[25] Schlaitz AL , Thompson J , Wong CC , Yates JR 3rd , Heald R . REEP3/4 ensure endoplasmic reticulum clearance from metaphase chromatin and proper nuclear envelope architecture. Dev Cell. 2013;26:315–23. 23911198 10.1016/j.devcel.2013.06.016 PMC3745822

[26] Wei H , Yan S , Hui Y , Liu Y , Guo H , Li Q , et al. CircFAT1 promotes hepatocellular carcinoma progression via miR‐30a‐5p/REEP3 pathway. J Cell Mol Med. 2020;24:14561–70. 33179443 10.1111/jcmm.16085 PMC7754024

[27] Nowick K , Stubbs L . Lineage‐specific transcription factors and the evolution of gene regulatory networks. Brief Funct Genomics. 2010;9:65–78. 20081217 10.1093/bfgp/elp056 PMC3096533

[28] Hamilton AT , Huntley S , Tran‐Gyamfi M , Baggott DM , Gordon L , Stubbs L . Evolutionary expansion and divergence in the ZNF91 subfamily of primate‐specific zinc finger genes. Genome Res. 2006;16:584–94. 16606703 10.1101/gr.4843906 PMC1457049

[29] Brass AL , Dykxhoorn DM , Benita Y , Yan N , Engelman A , Xavier RJ , et al. Identification of host proteins required for HIV infection through a functional genomic screen. Science. 2008;319:921–6. 18187620 10.1126/science.1152725

[30] Deng W , Wang L , Xiong Y , Li J , Wang Y , Shi T , et al. The novel secretory protein CGREF1 inhibits the activation of AP‐1 transcriptional activity and cell proliferation. Int J Biochem Cell Biol. 2015;65:32–9. 26022276 10.1016/j.biocel.2015.05.019

[31] Mang T , Kleinschmidt‐Doerr K , Ploeger F , Schoenemann A , Lindemann S , Gigout A . BMPR1A is necessary for chondrogenesis and osteogenesis, whereas BMPR1B prevents hypertrophic differentiation. J Cell Sci. 2020;133:jcs246934. 32764110 10.1242/jcs.246934

[32] Tian H , Zhou T , Chen H , Li C , Jiang Z , Lao L , et al. Bone morphogenetic protein‐2 promotes osteosarcoma growth by promoting epithelial‐mesenchymal transition (EMT) through the Wnt/β‐catenin signaling pathway. J Orthop Res. 2019;37:1638–48. 30737824 10.1002/jor.24244

[33] Jiang ZH , Peng J , Yang HL , Fu XL , Wang JZ , Liu L , et al. Upregulation and biological function of transmembrane protein 119 in osteosarcoma. Exp Mol Med. 2017;49:e329. 28496199 10.1038/emm.2017.41 PMC5454443

[34] Yu S , Guo J , Sun Z , Lin C , Tao H , Zhang Q , et al. BMP2‐dependent gene regulatory network analysis reveals Klf4 as a novel transcription factor of osteoblast differentiation. Cell Death Dis. 2021;12:197. 33608506 10.1038/s41419-021-03480-7 PMC7895980

[35] Li H , Han X , Yang S , Wang Y , Dong Y , Tang T . FOXP1 drives osteosarcoma development by repressing P21 and RB transcription downstream of P53. Oncogene. 2021;40:2785–802. 33716296 10.1038/s41388-021-01742-4

[36] Almada AE , Horwitz N , Price FD , Gonzalez AE , Ko M , Bolukbasi OV , et al. FOS licenses early events in stem cell activation driving skeletal muscle regeneration. Cell Rep. 2021;34:108656. 33503437 10.1016/j.celrep.2020.108656 PMC9112118

[37] Tan J , Liang H , Yang B , Zhu S , Wu G , Li L , et al. Identification and analysis of three hub prognostic genes related to osteosarcoma metastasis. J Oncol. 2021;2021:6646459. 33564309 10.1155/2021/6646459 PMC7867449

[38] Stanley A , Heo SJ , Mauck RL , Mourkioti F , Shore EM . Elevated BMP and mechanical signaling through YAP1/RhoA poises FOP mesenchymal progenitors for osteogenesis. J Bone Miner Res. 2019;34:1894–909. 31107558 10.1002/jbmr.3760 PMC7209824

